# T-Cell Accumulation in the Hypertensive Brain: A Role for Sphingosine-1-Phosphate-Mediated Chemotaxis

**DOI:** 10.3390/ijms20030537

**Published:** 2019-01-28

**Authors:** Nicholas Don-Doncow, Lotte Vanherle, Yun Zhang, Anja Meissner

**Affiliations:** 1 Department of Experimental Science, Lund University, 22 184 Lund, Sweden; nicholas.don-doncow@med.lu.se (N.D.-D.); lotte.vanherle.8386@med.lu.se (L.V.); yun.zhang@med.lu.se (Y.Z.); 2 Wallenberg Center for Molecular Medicine, Lund University, 22 184 Lund, Sweden

**Keywords:** hypertension, inflammation, sphingosine-1-phosphate, T-cell chemotaxis, cognitive impairment

## Abstract

Hypertension is considered the major modifiable risk factor for the development of cognitive impairment. Because increased blood pressure is often accompanied by an activation of the immune system, the concept of neuro-inflammation gained increasing attention in the field of hypertension-associated neurodegeneration. Particularly, hypertension-associated elevated circulating T-lymphocyte populations and target organ damage spurred the interest to understanding mechanisms leading to inflammation-associated brain damage during hypertension. The present study describes sphingosine-1-phosphate (S1P) as major contributor to T-cell chemotaxis to the brain during hypertension-associated neuro-inflammation and cognitive impairment. Using Western blotting, flow cytometry and mass spectrometry approaches, we show that hypertension stimulates a sphingosine kinase 1 (SphK1)-dependent increase of cerebral S1P concentrations in a mouse model of angiotensin II (AngII)-induced hypertension. The development of a distinct S1P gradient between circulating blood and brain tissue associates to elevated CD3+ T-cell numbers in the brain. Inhibition of S1P_1_-guided T-cell chemotaxis with the S1P receptor modulator FTY720 protects from augmentation of brain CD3 expression and the development of memory deficits in hypertensive WT mice. In conclusion, our data highlight a new approach to the understanding of hypertension-associated inflammation in degenerative processes of the brain during disease progression.

## 1. Introduction

Hypertension is the leading cause of disease burden in the developed world and a major modifiable risk factor for the development of several degenerative conditions in target organs [1,2]. Pathological changes in blood pressure (BP) have been directly linked to cognitive decline and initiated controversial discussions about anti-hypertensive medication as a potential therapeutic strategy for cognitive impairment emanating from hypertension. Although a meta-analysis of longitudinal studies confirmed the efficacy of anti-hypertensive treatment in reducing the risk of developing cognitive deficits, such treatment failed to reverse already established hypertension-associated cognitive dysfunction [3]. Due to the scarcity of mechanistic understanding regarding disease development, the best strategy for slowing or reversing cognitive decline resulting from hypertension is yet to be found.

In recent years, it became apparent that hypertension associates to an activation of the immune system with a critical involvement of T-lymphocytes in its pathogenesis [4,5,6,7,8]. Such chronic inflammation not only contributes to progression of hypertension [6,7,8] but also unfavourably affects target organs, including the brain [9,10,11,12]. Recently, a critical link between neuro-inflammation and cognitive dysfunction emerged as inflammation negatively affects brain function by dysregulating gene expression [13], altering neuronal function [14] and impairing neurogenesis [15]. Besides the existing association between elevated levels of pro-inflammatory cytokines such as Interleukin (IL)-6 or C reactive protein and cognitive impairment [16,17], studies have shown an augmentation of circulating T-cell numbers in patients with various neurodegenerative diseases [18] and moreover, an infiltration of T-lymphocytes into the brain of patients with Parkinson’s and Alzheimer’s disease [19,20,21]. Although these studies point to a critical involvement for T-cell infiltration to increasing disease burden and describe potential direct detrimental effects on cognitive function, the mechanism by which T-lymphocytes home to the brain remains to be determined. Similarly, the exact interplay between immune system activation during hypertension and degenerative consequences of hypertension in the brain is still elusive.

Growing evidence supports a critical role for the bioactive phospholipid sphingosine-1-phosphate (S1P) and its signalling axis not only in the pathogenesis of hypertension [6,7,22] but also in immune cell activation and trafficking (reviewed in Reference [6]). In response to a variety of stimuli, S1P is generated by two kinases (Sphk1 and Sphk2) and mediates its specific responses via different G-protein coupled receptors (S1P_1–5_) in a cell-type dependent fashion. In the immune system, S1P acts as an important chemotactic substance that critically modulates lymphocyte egress and homing [7,23,24,25]. Such S1P-governed chemotaxis requires specific S1P receptor expression on immune cells and has led to the development of several S1P receptor modulators, one of which is FDA approved for clinical applications in multiple sclerosis therapy: Fingolimod (FTY720), an S1P analogue that promotes an agonistic activation and subsequent internalization of S1P_1_ on lymphocytes, leads to lymphopenia since deficient S1P_1_ surface expression blocks S1P-governed lymphocyte egress from secondary lymphoid tissue (reviewed in Reference [26]). In a recent experimental undertaking, we describe a seminal S1P-mediated mechanism for T-cell responses critically involved in the development of experimental hypertension. In a murine model of Angiotensin II (AngII)-induced hypertension, increased BP associated to elevated circulating S1P levels that governs T-cell mobilization from the secondary lymphoid tissue. The same study showed the efficacy of fingolimod administration in preventing the onset of hypertension by inhibiting the egress of T-lymphocytes from secondary lymphoid tissues. Besides its trafficking effects, S1P modulates cerebrovascular responses [7,27,28] and together with its generating enzyme SphK1 it is suggested to be involved in the production of the pro-inflammatory cytokine IL-17A in microglia [29]. Both, IL-17 secreting T-cells and cerebrovascular alterations are detrimental to cognitive function [1,2,30,31,32,33].

In this study, we aim to expand upon findings that define S1P as an important chemotactic substance for T-cell trafficking during hypertension by exploring the hypothesis that AngII-induced generation of S1P in the brain of hypertensive mice may be involved in T-cell infiltration into the brain and thus, the development of neuro-inflammation and eventually cognitive dysfunction.

## 2. Results

### 2.1. Hypertension-Associated Cognitive Deficits Link to Elevated S1P Levels in the Brain

**Figure 1:**
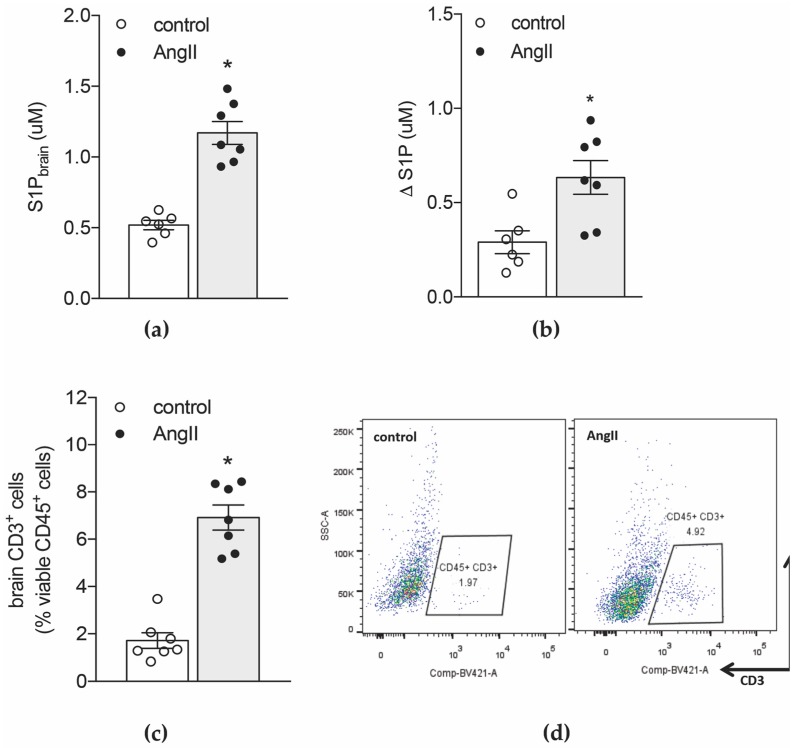
Hypertension links to elevated cerebral S1P levels and an accumulation of CD3+ T-cells in the brain. (**a**) Mass spectrometry analysis of S1P levels in brain tissue of normotensive and hypertensive WT mice. (**b**) Concentration difference between brain and plasma S1P levels in normotensive and hypertensive WT mice expressed as delta values (Δ [S1P]). (**c**) Percentage of CD3+ leukocytes in the brain of normotensive and hypertensive WT mice assessed in a FACS-based approach. (**d**) Representative dot blots showing CD45+ CD3+ cells in brain tissue of normotensive and hypertensive WT mice. Data expressed as mean ± SEM; *n* = 6–7 per group * *p* < 0.05 after single unpaired comparisons.

We recently reported that memory deficits in a mouse model of hypertension were not only accompanied by alterations in vascular structure and brain blood flow but also elevated transcript levels of pro-inflammatory cytokines and chemokines [2]. Amongst other potent chemoattractants [2], brain tissue of hypertensive mice presented with higher concentrations of S1P compared to normotensive controls (Figure 1a). Although hypertension also links to an increase in S1P plasma concentrations [7], the calculation of the difference between the concentration of brain S1P and plasma S1P (Δ [S1P]) revealed significantly higher values in the hypertensive group (Figure 1b), suggesting that hypertension promotes the development of stronger S1P gradients between brain tissue and circulating blood. Thus, S1P-governed immune cell chemotaxis might contribute to the elevated proportion of CD3+ T-cells we detected in hypertensive compared to normotensive brains using a FACS-based approached (Figure 1c,d).

### 2.2. Genetic Depletion of S1P Generating Enzyme SphK1 Protects from AngII-Induced Increases in Brain S1P Levels and Memory Deficits

**Figure 2:**
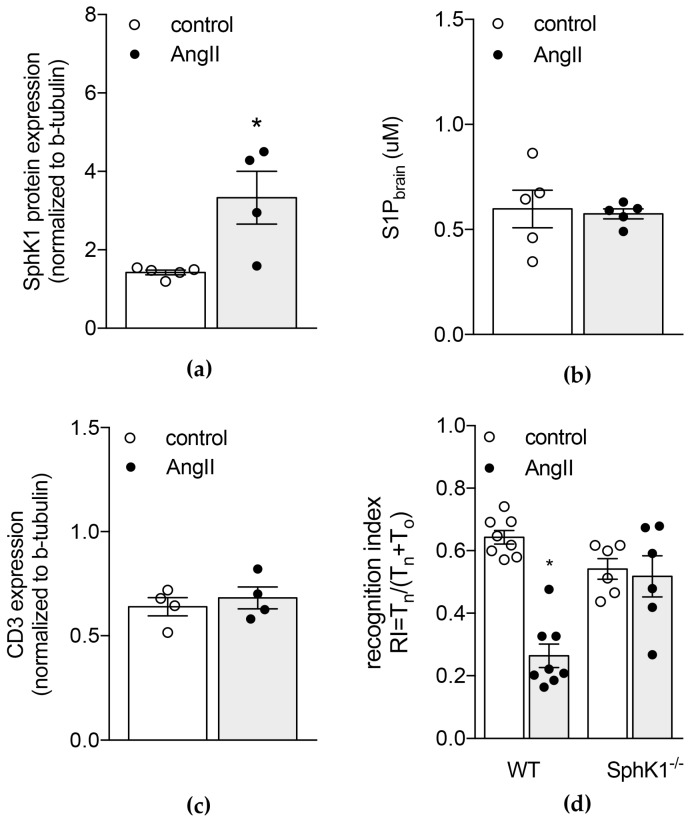
Genetic depletion of SphK1 protects from AngII-induced increases in brain S1P levels and memory deficits. (**a**) Western blot analysis of SphK1 protein expression in the brain of normotensive and hypertensive WT mice (corresponding Western blot images can be found in Figure S1b). (**b**) Mass spectrometry analysis of brain S1P levels in SphK1-deficient mice chronically perfused with AngII or saline. (**c**) Western blot analysis of brain CD3 protein expression in saline or AngII-treated SphK1-deficient mice (corresponding Western blot images can be found in Figure S3b). (**d**) Memory function of saline or AngII-treated SphK1-deficient and WT mice assessed in a NOR task with 24 h delay interval. Data expressed as mean ± SEM; *n* = 4–8 per group * *p* < 0.05 after single unpaired comparisons.

In order to investigate the link between AngII-induced hypertension and changes in brain S1P concentrations, we first verified expression levels of S1P generating enzymes in brain tissue of normotensive and hypertensive WT mice. In hypertensive WT mice, increased brain S1P levels were associated with augmented *sphk1* mRNA (Figure S1) and protein expression (Figure 2a). In contrast, *sphk2* mRNA expression was not affected by AngII-induced hypertension (Figure S2).

We next confirmed the SphK1-mediated S1P response to chronic AngII perfusion by assessing brain S1P concentrations in mice lacking SphK1. Here, no difference was observed between control and AngII-treated groups (Figure 2b). The genetic depletion of *sphk1* (0.002 ± 0.001 in SphK1^−/−^ mice vs. 1 ± 0.083; *p* = 0.0357 in WT control mice; Table S3) resulted in a higher baseline expression of *sphk2* mRNA compared to WT control mice (1.875 ± 0.352 vs. 1 ± 0.058; *p* = 0.0447; Table S3). Chronic perfusion with AngII did not affect *sphk2* mRNA (Table S3 and Figure S2, respectively). Together, these data are suggestive of an SphK1-mediated S1P response to AngII in the brain.

Different from WT mice, SphK1-deficient mice were protected from an AngII-associated increase in circulating T-cell counts (Table S4) and similarly, AngII treatment failed to increase brain CD3 protein expression in SphK1-deficient mice (Figure 2c). Furthermore, we observed a significantly lower CD3 protein expression in SphK1^−/−^ mice after chronic AngII treatment compared to that observed in hypertensive WT mice (Figure S3). Moreover, SphK1^−/−^ mice showed no signs of neuro-inflammation in response to AngII treatment: brain transcripts of pro-inflammatory cytokines such as tumour necrosis factor alpha (*Tnfa*) and IL-1β (*Il1b*) as well as endothelial activation markers such as von Willebrand factor (*Vwf*) and P-selectin (*Selp*) did not differ between the groups (Table 1). Compared to hypertensive WT mice, chronic AngII treatment resulted in significantly lower expression levels of *Tnfa*, *Il1b* and *Selp* when SphK1 was genetically depleted (Table 1). Interestingly, the absence of SphK1 presented with markedly elevated *Vwf* expression levels at baseline (5.21-fold increase compared to WT control; *p* = 0.0407; Table S5). Following AngII treatment, no significant difference was observed in comparison to hypertensive WT mice (Table 1).

Different from WT mice, AngII treatment failed to induce memory deficits in SphK1^−/−^ mice (Figure 2d).

### 2.3. Inhibiting S1P Chemotaxis Protects from Hypertension-Associated Cognitive Impairment

**Figure 3:**
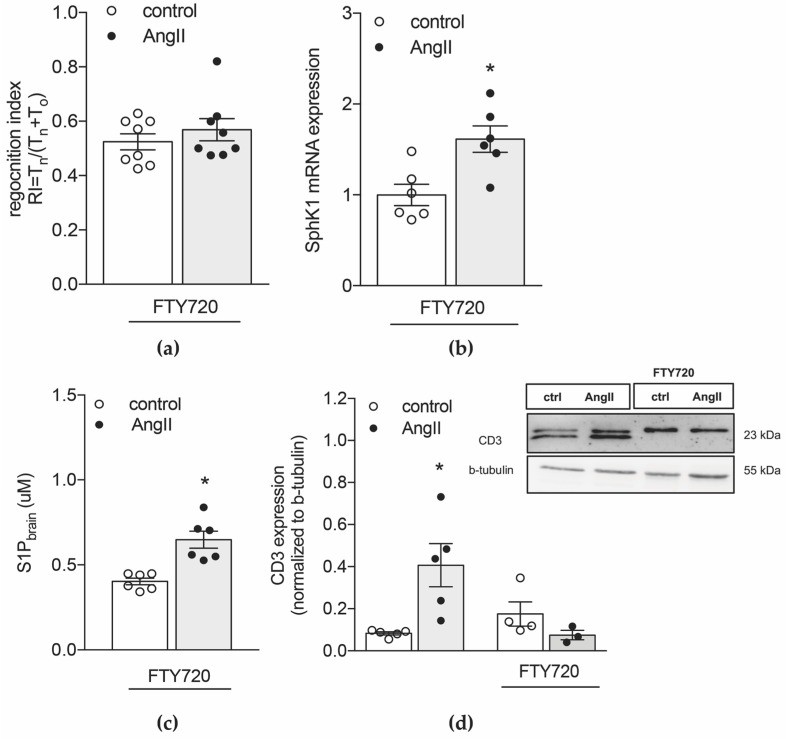
Inhibiting S1P chemotaxis protects from hypertension-associated cognitive impairment. (**a**) Memory function was assessed in a novel object recognition task with 24 h delay interval in mice treated with 1mg/kg BW FTY720 for two constitutive weeks. (**b**) qPCR analysis of *sphk1* mRNA expression in normotensive and hypertensive mice treated with 1mg/kg BW FTY720. (**c**) Mass spectrometry analysis of S1P levels in the brain of normotensive and hypertensive WT mice treated with FTY720. (**d**) Western blot analysis of brain CD3 protein expression in normotensive and hypertensive WT mice with or without FTY720 treatment. Data expressed as mean ± SEM; *n* = 6–8 per group * *p* < 0.05 after single unpaired comparisons.

Because SphK1^−/−^ mice show a blunted BP response to AngII (Figure S4), which might influence the cognitive readouts we set out to test our hypothesis in hypertensive WT mice where we pharmacologically inhibited T-cell mobilization from secondary lymphoid tissue using fingolimod (FTY720) [34]. Therefore, we treated hypertensive WT mice and normotensive controls with FTY720 (1mg/kg BW) for two constitutive weeks before memory function, immune cell status and S1P levels were determined. Despite inducing profound lymphopenia (Table S4), FTY720 did not affect BP levels [7] but prevented the development of memory deficits (Figure 3a). Similar to WT mice, AngII treatment induced significantly augmented *sphk1* mRNA and protein expression (Figure 3b and Figure S5, respectively) and linked to elevated S1P levels in brain (Figure 3c). Interestingly, FTY720 treatment resulted in significantly higher baseline *sphk2* mRNA levels in the brain compared to untreated normotensive WT mice (1.571 ± 0.097 vs. 1 ± 0.038; *p* = 0.0286; Table S5). This, however, did not translate to higher cerebral S1P concentrations (0.4026 ± 0.0502 in normotensive WT mice treated with FTY720 vs. 0.5376 ± 0.0333 in normotensive WT mice; *p* = 0.5969). Different from *sphk1* expression (Figure 3b), AngII treatment did not affect *sphk2* expressions following FTY720 treatment (Table S3), suggesting an SphK1-mediated increase of cerebral S1P concentrations in response to AngII. The calculated difference between S1P concentrations in brain and plasma revealed higher Δ [S1P] values in the hypertensive group (Figure S6) suggestive of S1P gradients similar to untreated hypertensive WT mice. Due to the lymphopenia induced by FTY720 (Table S4), CD3 brain expression did not change between normotensive and hypertensive mice (Figure 3d). Similar findings were obtained in lymphocyte-deficient Rag2^−/−^ mice where AngII treatment associated to an elevation of S1P levels in the brain (Figure S7) whereas CD3 brain expression remained unaltered (Figure S8).

S1P-guided chemotaxis in T-cells is mainly mediated by S1P_1_ surface expression [6]. Together with our findings showing increased S1P concentrations in the hypertensive brain, markedly higher numbers of circulating S1P_1_+ CD3+ T-cells observed in hypertensive WT mice (Table 2) suggest an involvement of S1P-mediated T-cell trafficking to the brain during hypertension. Because S1P_1_ is internalized upon exposure to high S1P concentrations [35] it is not surprising that the percentage of S1P_1_+ T-cells in brain tissue did not differ between normotensive and hypertensive WT mice (Figure S9). Unaltered circulating S1P_1_+ T-cell numbers in AngII-treated SphK1^−/−^ mice (Table 2) that lack an S1P gradient between blood and brain are supportive of this hypothesis. When targeting S1P receptors with FTY720, both normotensive and hypertensive WT mice presented with low numbers of circulating S1P_1_+ T-cell (Table 2). Subsequently, FTY720-induced lymphopenia diminished hypertension-associated increases in brain CD3 expression despite elevated brain S1P concentrations.

## 3. Discussion

The present study describes S1P-goverened T-cell chemotaxis to the brain as a potential contributing factor to hypertension-associated neuro-inflammation and cognitive impairment. Our data confirm that hypertension stimulates an SphK1-dependent increase of S1P concentrations in the brain, promoting the development of a distinct S1P gradient between brain tissue and circulating blood that in turn, might govern S1P_1_-guided T-cell chemotaxis to the hypertensive brain.

The concept of immune cell infiltration to the brain gained more and more attention in the field of neurodegenerative disease research in recent years. It became apparent that T-lymphocytes not only play a major role in neurodegenerative processes during auto-immune diseases such as multiple sclerosis [33,36,37,38] but also in disease progression of Alzheimer’s and Parkinson’s disease (reviewed in Reference [18,39]. Similarly, the growing interest in understanding the pathogenic mechanisms leading to neuro-inflammation and cognitive dysfunction as a consequence of cardiovascular disease (CVD) disclosed apparent accumulation of CD3+ and CD4+ T-cells in the brain during several CVDs [2,33,38,40,41], which aligns with our findings presented in this study. Alterations of T-lymphocyte populations in the circulation in both classical neurodegenerative diseases, such as AD or PD [42] and neurodegeneration induced by hypertension [7,8,43] suggest a contributory role of T-cell-associated inflammation in disease development, progression and severity. To our knowledge, we are the first to show that blocking T-cell entry to the brain protects from the development of memory deficits in a mouse model of experimental hypertension. Although our study did not investigate a direct mechanistic link between T-cell infiltration and cognitive dysfunction, we convincingly provide evidence that T-cell trafficking to the brain plays an important role in neurodegenerative processes during AngII-induced hypertension.

The main barrier for immune cells to enter the brain, the blood brain barrier (BBB), has shown increased permeability during hypertension [2,44]. Together with augmented expression of adhesion molecules such as P-selectin and VCAM-1 [2], hypertension promotes an environment for enhanced peripheral immune cell entry to the brain. However, immune cells require chemotactic cues to migrate into tissue. Our results put forward the hypothesis of an S1P-governed T-cell infiltration to the hypertensive brain. S1P serves as a potent chemoattractant for a variety of immune cells, including T-lymphocytes [6,34,45,46]. Furthermore, S1P gradients between tissues critically drive lymphocyte egress and homing [6,33,47]. The delicate regulation of S1P gradients is thought to involve different S1P signalling components such as SphKs that synthesize S1P [48,49,50,51] or S1P catabolizing enzymes that control S1P levels within the primary and secondary lymphoid organs [52,53,54]. However, the contribution of the different enzymes to controlling tissue-specific S1P concentrations under pathological conditions is mostly elusive. Glial cells and neurons have been recognized as the main contributors to the local S1P pool in the brain [29,55,56], yet S1P produced by vascular smooth muscle cells [57] might also be supplying this local pool. Although the exact mechanisms leading to the augmented cerebral S1P concentrations we observe in our model of hypertension still remain subject of speculation, our results support the involvement of an SphK1-mediated S1P generation as mice lacking this enzyme were protected from the elevation of S1P levels in the brain despite a compensatory up-regulation of SphK2 expression. Our data furthermore suggest a rather direct effect of AngII in the augmented SphK1-mediated cerebral S1P production as Rag-deficient mice that are protected from high BP [43] show an elevation of brain S1P concentration similar to hypertensive WT mice. Because blood-borne AngII accumulates in the perivascular space of hypertensive mice as early as fourteen days post disease initiation [44], AngII might directly stimulate the SphK1-mediated S1P generation in cell types in close proximity to the perivascular space for instance, astrocytes or vascular smooth muscle cells. Another mechanism currently discussed involves the activation of SphK1 in microglia, which associates to the production of chemotactic substances and pro-inflammatory mediators [29,58] and hence, may promote the migration of peripheral immune cells such as T-lymphocytes to the brain (reviewed in Reference [59]). The mechanisms by which AngII may directly or indirectly activate microglia-specific SphK1 activation needs to be considered in future studies.

Increased S1P levels in inflamed tissue might persuade enhanced T-cell retention as T-cells internalize S1P_1_ upon exposure to high S1P concentrations [35]. In line with that, our study supports an S1P- S1P_1_-governed chemotaxis of T-cells to the hypertensive brain as hypertensive WT mice presented with a higher number of circulating S1P_1_+ T-cells compared to mice lacking the AngII-induced S1P response in the brain (SphK1^−/−^) or hypertensive WT mice in which T-cell mobilization was blocked with the S1P receptor modulator FTY720. Together with the absence of CD3 signal in the brain of SphK1^−/−^ and FTY-720-treated WT mice, comparable cerebral S1P_1_+ T-cell numbers between control and hypertensive WT mice strongly support a critical involvement of S1P-mediated T-cell infiltration into the brain during hypertension.

Besides immune cell chemotaxis, S1P exerts effects on other cell types in the brain. In neuronal cells for instance, S1P can stimulate proliferation or apoptosis dependent on their differentiation state [60,61], up-regulates glutamate secretion and enhances excitatory activity [62]. Microinjection of S1P into the healthy brain induced neuroglia activation evident by an augmentation of ionized calcium-binding adapter molecule (Iba)-1+ and glial fibrillary acidic protein (GFAP)+ cells [63]. We therefore cannot exclude the possibility that S1P directly impairs the function of brain resident cells long term and thus, contributes to cognitive dysfunction. In sensory neurons and motor neuron-like cells, S1P caused a rapid retraction of neurites and led to a growth cone collapse [64]. Interestingly, genes encoding the S1P signalling pathway were found to be enriched among genes associated with increases in lateral ventricular volume, a key feature of several neurological and psychiatric diseases [65]. These findings are suggestive of additional direct effects of elevated S1P levels on brain cells, which necessitate further investigation not only in the context of hypertension-induced neurodegenerative processes but also classical neurodegenerative diseases.

**Figure 4:**
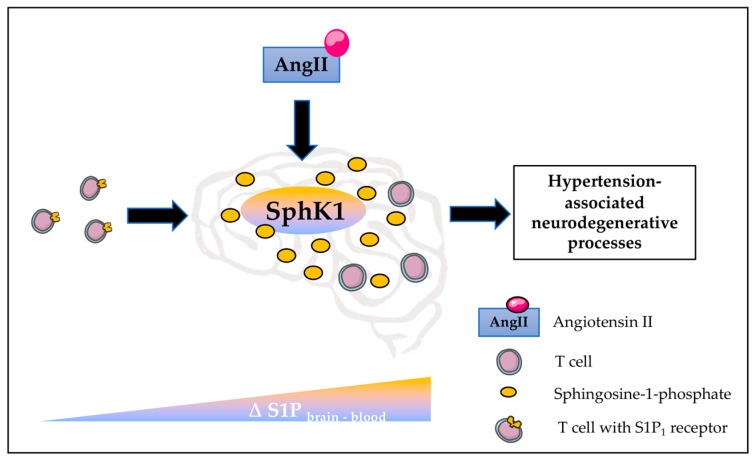
Central illustration. AngII stimulates an SphK1-mediated elevation of brain S1P concentrations, which establishes an S1P gradient between the brain and the circulating blood that allows S1P-governed T cell infiltration to the brain. AngII—angiotensin II, S1P—sphingosine-1-phosphate, S1P_1_—S1P receptor type 1.

Together, our findings suggest an SphK1-mediated elevation of cerebral S1P concentrations in response to AngII, which might be causative to S1P-governed attraction of T-cells to the hypertensive brain (Figure 4). Our herein presented results provide first evidence of S1P involvement in such inflammatory mechanisms that critically contribute to hypertension-associated degenerative processes in the brain. Thus, our findings unveil a new interpretation on how CVDs like hypertension might contribute to brain degeneration. Future experimental endeavours need to tackle the exact mechanisms underlying direct effects of S1P on brain resident cells and potential T-cell mediated contributions to neurodegeneration in the context of hypertension.

## 4. Materials and Methods

### 4.1. Materials

All chemical reagents and solutions were purchased from Fisher Scientific (Göteborg, Sweden), Saween & Werner (Limhamn, Sweden) or Sigma-Aldrich (Stockholm, Sweden) unless otherwise stated. Commercially available primary antibodies against CD3 (Abcam, Cambridge, UK), SphK1 (Nordic Biosite, Täby, Sweden) and β-tubulin (Sigma Aldrich, Stockholm, Sweden) were used for Western blotting. Primers for qPCR were purchased from Eurofins (Ebersberg, Germany).

### 4.2. Animals

This investigation conforms to the Guide for Care and Use of Laboratory Animals published by the European Union (Directive 2010/63/EU) and with the ARRIVE guidelines. All animal care and experimental protocols (5.8.18/12637/2017; 7143-18) were approved by the institutional animal ethics committee at Lund University and were conducted in accordance with European animal protection laws. Wild-type (WT) C57Bl/6N mice were obtained from Taconic (Ejby, Denmark) and housed in a conventional animal facility under standard conditions with a 12:12 h light-dark cycle and access to food (standard rodent diet) and water *ad libitum*. Mice with a body weight BW ≥25g were housed in groups of four to five in conventional transparent polycarbonate cages. In order to obey the rules for animal welfare, we designed experimental groups in a way that minimizes stress for the animals and guarantees maximal information using the lowest group size possible when calculated with a type I error rate of α = 0.05 (5%) and Power of 1-β > 0.8 (80%) based on preliminary experiments.

### 4.3. Hypertension Model

Hypertension was induced using angiotensin II (AngII)-releasing osmotic mini pumps as previously described [7]. In brief, WT or transgenic male and female mice (12 weeks old, gender equally distributed to all experimental groups) were anesthetized with isoflurane (2.5% at 1.5 L/min oxygen) for subcutaneous implantation of osmotic mini-pumps (Alzet-1004; AgnThos, Lindingö, Sweden) containing AngII (1 ug/kg per min at an infusion rate of 11 µL/h) or an equivalent volume of vehicle (saline). Systolic BP was measured in conscious mice using tail-cuff plethysmography (EMKA Technologies, France) starting seven days before pump implantation after an initial training period of seven days. Experimental groups were the following: WT mice that received AngII (*n* = 14) and respective controls (*n* = 14); SphK1^−/−^ mice that received AngII (*n* = 10–12) and respective controls (*n* = 10–12); Rag2^−/−^ mice that received AngII (*n* = 6) and Rag2^−/−^ control mice (*n* = 6).

Fingolimod (1 mg/kg BW in Saline, i.p.) or saline was injected every 48 h after an initial daily injection period of three days starting two weeks after implantation of AngII-filled pumps (*n* = 12 per group).

Four weeks after pump implantation, mice were anaesthetized (isoflurane 2.5% at 1.5 L/min oxygen) for whole blood collection before euthanasia through cervical dislocation.

### 4.4. Novel Object Recognition (NOR)

As previously described [2,12], a NOR task was employed to assess non-spatial memory components. Briefly, mice were habituated to the testing arena for 8 min over a period of 3 days. On test day, each mouse was exposed to two objects for 5 min. 24 h later, mice were re-exposed to one object from the original test pair and to a novel object. The movements of the animal were video tracked with the computer software AnyMaze^®^ (Stoelting, Dublin, Ireland). A recognition index (RI) as the main index of retention was calculated by the time spent investigating the novel object relative to the total object investigation (RI = *T*_Novel_/(*T*_Novel_ + *T*_Familiar_)). To ensure blinding behavioural assessment was performed after the animals had received codes that did not reveal the identity of the treatment.

### 4.5. Mass Spectrometry

S1P concentrations in plasma and brain tissue were determined with mass spectrometry using a TSQ Quantum Ultra-Triple quadrupole mass spectrometer equipped with an ESI probe and interfaced with an LC system. Brain tissue was homogenized in 1 M NaCl before 30 µM internal standard (IS; D-*erythro*-sphingosine-1-phosphate (C17 base) (Avanti Polar Lipids, (Delfzijl, The Netherlands), an equivalent volume of MeOH and 10% 6M HCl was added. Separation of the organic phase was carried out by centrifugation (1900× *g* for 3 min) after the addition of an equivalent volume of CHCl_3_. Following evaporation of CHCl_3_ in a vacuum concentrator, samples were suspended in 100 µL MeOH:CHCl_3_ (4:1) and stored at −80 °C until further usage. Further methodological details can be found in the supplementary information.

### 4.6. Fluorescence Activated Cell Sorting

Brain tissue was enzymatically digested and homogenized. After density separation using Percoll (GE Healthcare), pellets were reconstituted in FACS buffer (PBS, 2 mM EDTA, 2% FBS) and incubated with antibodies for 30 min (see Table S1 in data supplement for detailed list of antibodies). Red blood cells (RBCs) obtained after RBCs lysis were incubated with primary antibodies in FACS buffer for 30 min at 4 °C protected from light. After centrifugation, the supernatant was decanted, washed and re-suspended in FACS buffer. Data were acquired in a FACS LSRII (BD Biosciences) using FacsDiva software (BD Biosciences). Cells were plotted on forward versus side scatter and single cells were gated on FSC-A versus FSC-H linearity. Flow-Count Fluorospheres (Life Technologies, Göteborg, Sweden) were used for absolute quantification.

### 4.7. Western Blotting and qPCR

Standard biochemical procedures were utilized for experiments involving reverse transcription polymerase chain reaction, quantitative PCR and Western blotting. Methodological details are provided in the supplement.

### 4.8. Statistical Analysis

All data are expressed as mean ± SEM, where n is the number of animals. For comparison of two groups a Mann-Whitney test was utilized. For comparisons of multiple groups, Kruskal-Wallis analyses with Dunn’s post hoc testing were performed. Differences were considered significant at error probabilities of *p* ≤ 0.05.

## Figures and Tables

**Table 1:** Chemokine and cytokine transcript levels in brain tissue of SphK1^−/−^ mice. qPCR-based assessment of pro-inflammatory chemokines and cytokines and endothelial activation markers in brain tissue of SphK1^−/−^ mice after chronic AngII or saline perfusion. Comparisons to hypertensive WT mice (WT AngII) are indicated as fold changes. Data expressed as mean ± SEM; *n* = 6; *p* values calculated for single unpaired comparisons.

	Control	AngII	*p*-Value	SphK1^−/−^ AngII vs. WT AngII (Fold Change)	*p*-Value
*Vcam1*	1.652 ± 0.227	1.870 ± 0.189	0.841	0.48	0.057
*Tnfa*	0.827 ± 0.267	0.431 ± 0.112	0.286	0.25	0.009
*Il1b*	1.246 ± 0.153	1.346 ± 0.247	0.841	0.29	0.037
*Vwf*	4.830 ± 1.672	2.859 ± 0.642	0.556	0.66	>0.999
*Selp*	0.647 ± 0.192	0.429 ± 0.187	0.413	0.19	0.003

**Table 2:** Number of circulating S1P_1_+ CD3+ T-cells. Flow cytometry-based assessment of circulating S1P_1_+ CD3+ T-cell numbers in blood samples of WT, SphK1^−/−^ and FTY720-treated WT mice after chronic AngII or saline perfusion. Data expressed as mean ± SEM; *n* = 10 for WT and *n* = 6 for SphK1^−/−^ and FTY20-treated WT groups; * *p* < 0.05 after single unpaired comparisons.

	Control	AngII	*p*-Value
WT	388.3 ± 87.1	3069.1 ± 764.9 *	0.0006
SphK1^−/−^	709.2 ± 213.2	433.7 ± 37.9	0.3939
WT + FTY720	372.2 ± 123.8	158.7 ± 59.2	0.1241

## Supplementary Materials

The following are available online at http://www.mdpi.com/1422-0067/20/3/537/s1.

## Abbreviations

AngII	Angiotensin II
BBB	Blood brain barrier
BP	Blood pressure
CD3	Cluster of differentiation 3
CD45	Cluster of differentiation 45
CVD	Cardiovascular disease
FACS	Fluorescence activated cell sorting
FTY720	Fingolimod
GFAP	Glial fibrillary acidic protein
Iba	Ionized calcium-binding adapter molecule
IL	Interleukin
NOR	Novel object recognition
RI	Recognition index
S1P	Sphingosine-1-phosphate
SphK1	Sphingosine kinase 1
SphK2	Sphingosine kinase 2
S1P_1_	Sphingosine receptor type 1
TNFA	Tumour necrosis factor alpha
VWF	Van Willebrand factor
VCAM-1	Vascular cell adhesion protein 1
WT	Wild type
