# Supplementary material for: T-Cell Accumulation in the Hypertensive Brain: A Role for Sphingosine-1-Phosphate-Mediated Chemotaxis
Source: Int J Mol Sci. 2019 Jan 28;20(3):537. doi: 10.3390/ijms20030537 (PMC6386943; doi:10.3390/ijms20030537)
Supplement: Supplementary file 1 [file ijms-20-00537-s001.pdf]

Sample preparation for FACS analysis**:** Whole blood samples were collected from the portal vein of anaesthetized mice (n = 6–10 per group) using a heparin-coated syringe (Sarstedt, Helsingborg, Sweden) and transferred to EDTA-coated collection tubes (Sarstedt, Helsingborg, Sweden). For plasma extraction, samples were centrifuged for 20 min (5000 rpm, 4 °C) and the supernatant was used in mass spectrometry. For fluorescence activated cell sorting (FACS) analysis, whole blood samples from EDTA collection tubes were directly processed using a standardized red blood cell (RBCs) lysis protocol. Briefly, RBCs were lysed protected from light in freshly prepared 1X RBC lysis buffer (150 mM NH_4Cl, 10 mM $KHCO_3$, 0.1 mM EDTA) for 10 min at room temperature. To stop the reaction, an equal amount of 1× PBS was added before centrifuging the samples at 300× *g* at room temperature. The resulting cell pellet was washed and re-suspended in the appropriate FACS buffer (PBS, 2 mM EDTA, 2% FBS) containing Fc-Block (1/200; Clone 2.4G2; BD Pharmingen) for further cell surface staining. 30 min of antibody incubation was conducted at 4 °C under the protection from light. Antibodies used for cell staining are listed in Table A1.

Table S1. Antibodies utilized in flow cytometry experiments for whole blood and brain tissue.

Antigen	Fluorophore	Clone	Supplier
Live/Dead Aqua	Emission max at 512 nm		Invitrogen
CD45	AF700	30-F11	R&D Systems
CD45R	AF488	RA3-6B2	R&D Systems
CD3	efluor450	17A2	eBioscience
CD11b	PE-Texas Red	M10/70.15	Invitrogen
S1P1	PE	713412	R&D Systems

Mass spectrometric measurement of S1P concentrations**:** The two hemispheres of the brain from half the animals per group were separated and distributed to either mRNA-protein extraction or mass spectrometry. For the isolation of the lipid fraction and mass spectrometric determination of S1P levels, plasma samples (*n* = 6–8) were diluted in 1ml extraction buffer (Isopropanol:water:ethyl acetate (7:1:12) and 30 µM (IS; D-*erythro*-sphingosine-1-phosphate) as internal standard (IS), mixed and centrifuged at 5000 rpm for 3 min. The collected supernatant was directly used for mass spectrometry.

Samples were analysed by LC-MS/MS on an API 3000 (AB Sciex) triple quadrupole mass spectrometer integrated on an Acquity UPLC system (Waters Corporation, Milford, MA, USA). A Kinetex (Phenomenex, Torrance, CA, USA) 2.6 µm XB C18 100 Å 50 × 2.00 mm column was used as stationary phase. The column temperature was set at 60 °C. The Mobile phases consisted of (A) 75% methanol, 0.1% formic acid and 5 mM ammonium formate and (B) 100% methanol, 0.1% formic acid and 5 mM ammonium formate. The gradient elution profile was as follows: 0 min 100% A, from 0 min to 2 min the % A decreased from 100% to 0 % and was kept at 0% until 5.5 min, when it was returned to initial conditions at 5.6 min until 6 min. Flow was set at 0.6 mL/min. Analyte was detected in positive electrospray ionization (ESI) mode. The ion spray voltage was set at 5000 Volts and source temperature at 400 °C. Data was acquired in multiple reaction monitoring (MRM) mode. Transitions selected were 380.1/264.4 for S1P and 387.3/271.5 for the internal standard. Results were integrated and calculated using linear regression by the Analyst 1.4.2 software (AB Sciex).

Quantitative real-time PCR (qRT-PCR)**:** The two hemispheres of the brain from half the animals per group were separated and distributed to either mRNA-protein extraction or mass spectrometry. RNA was isolated from brain tissue (*n* = 4–8 per group) using a Trizol® method according to the manufacturer's protocol. As per instructions using a "High Capacity Reverse Transcriptase Kit" kit (Applied Biosystems, Göteborg, Sweden), 1 µg of total RNA was reverse transcribed with random hexamer primers. The resulting cDNA was diluted to a final volume of 500 µL and subsequently used as a template for qRT-PCR reactions. qRT-PCR was performed in triplicates using *Power* SYBR® Green PCR Master Mix (Applied Biosystems, Göteborg, Sweden) according to the manufacturer's instructions. Each reaction comprised 1ng cDNA, 3ul master mix and 0.2 uM final concentration of each primer. Cycling and detection were carried out using a CFX Connect™ Real-Time PCR Detection

System and data quantified using Sequence CFX Manager™ Software (Biorad, Göteborg, Sweden). qRT-PCR was performed for a total of 40 cycles (95 °C 15 s, 60 °C 60 s) followed by a dissociation stage. All primers (see Table A2 for primer sequences) were optimized prior first usage by assessing the optimal annealing temperature using a temperature gradient. Specificity was monitored using melt-curve validation. The efficiency, reproducibility and dynamic range of a qPCR assay were determined by constructing a standard curve using serial dilutions of pooled cDNA representative of each sample to be analysed and water as negative control. A standard curve was constructed by plotting the log of the dilution factor against the CT value obtained during amplification of each dilution (performed in duplicates). The qPCR assay was considered optimal with a coefficient of determination (R^2) value >0.950 and similar CT values of the replicates. All data were normalized to L14 and quantification was carried out via the absolute method using standard curves. Test samples were assayed with the standards in the same experimental run. The standard curve constructed from the diluted standard template was used to determine the target quantity in the samples by interpolation.

Table S2. Primer sequences used in qPCR experiments.

Target	Forward primer	Reverse primer	Tm
sphk1	5′-GGTGAATGGGCTAATGGAACG-3′	5′-CTGCTCGTACCCAGCATAGTG-3′	60 °C
sphk2	5′-CACGGCGAGTTTGGTTCCTA-3′	5′-CTTCTGGCTTTGGGCGTAGT-3′	60 °C
L14	5′-GGCTTTAGTGGATGGACCCT-3′	5′-ATTGATATCCGCCTTCTCCC-3′	60 °C
Vcam1	5′-TGCCGAGCTAAATTACACATTG-3′	5′-CCTTGTGGAGGGATGTACAGA-3′	60 °C
Vwf	5′-CTTCTGTACGCCTCAGCTATG-3′	5′-GCCGTTGTAATTCCCACACAAG-3′	60 °C
Selp	5′-CATCTGGTTCAGTGCTTTGATCT-3′	5′-ACCCGTGAGTTATTCCATGAGT-3′	60 °C
Il1b	5′-GAA GAG CCC ATC CTC TGT GA-3′	5′-TTC ATC TCG GAG CCT GTA GTG-3′	60 °C
Tnfa	5′-CCC TCA CAC TCA CAA ACC ACC-3′	5′-GCC TTG TCC CTT GAA GAG AAC C-3′	60 °C

Western Blotting: Protein isolation was performed in a sequential precipitation of RNA, DNA and protein from a single sample using the Trizol® method. Following RNA and DNA precipitation, protein was extracted from the phenol-ethanol supernatant by isopropanol precipitation according to the manufacturer's instructions. After removal of impurities and solubilization, protein concentration was determined by PIERCE BCA protein assay (Fisher Scientific, Göteborg, Sweden). Western blotting was carried out according to standard protocols. Briefly, samples were heated for 10 min at 95 °C in sample buffer (2.0 mL 1M Tris-HCl, pH 6.8, 2% SDS, 10% glycerol, 1% 14.7 M β-mercaptoethanol 12.5 mM EDTA, 0.02% bromophenol blue). Proteins were then separated on 12–15% Bis-acrylamid mini gels containing 0.1% 2,2,2-Trichloroethanol for subsequent visualization of protein loading using a Chemidoc MP imaging system (Biorad, Stockholm, Sweden). Following protein transfer onto PVDF membranes (Biorad, Stockholm, Sweden), transfer efficiency was checked using a Chemidoc MP imaging system. The membranes were blocked for 60 min in 1% bovine serum albumin (in phosphate-buffered saline containing 1% Tween 20 (PBST); 137 mM NaCl, 2.7 mM KCl, 10 mM Na_2HPO_4, 1.76 mM K_2HPO_4; pH 7.4) and sequentially incubated with the primary and secondary antibodies. A 1:1000 dilution for the primary antibody (CD3 and SphK1 (Abcam, Cambridge, UK), β-tubulin (Sigma Aldrich, Stockholm, Sweden)) and 1:10,000 for the HRP-labelled secondary antibody (anti-rabbit IgG or anti-mouse IgG BioNordica, Stockholm, Sweden) were utilized. All antibodies were diluted in 1% bovine serum albumin in PBST. A standard chemiluminescence procedure was used to visualize protein binding in a Chemidoc MP imaging system. The images were evaluated densitometrically using Image Lab 6.0.1 (Biorad, Stockholm, Sweden) or "Image J" software (1.48v, http://imagej.nih.gov/ij).

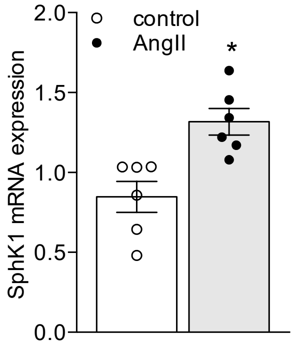

(a)

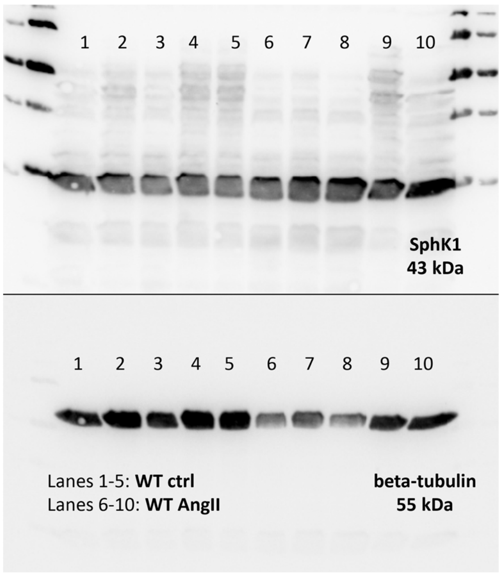

(b)

Figure S1. SphK1 expression increases in brain tissue of hypertensive WT mice. (**a**) qPCR analysis of *sphk1* mRNA expression in whole brain tissue from normotensive and hypertensive WT mice. Data expressed as mean ± SEM; $n = 6$ per group * $p < 0.05$ after single unpaired comparisons. (**b**) Representative Western blot image illustrating AngII-induced up-regulation of SphK1 protein expression in brain tissue lysates of normotensive and hypertensive WT mice.

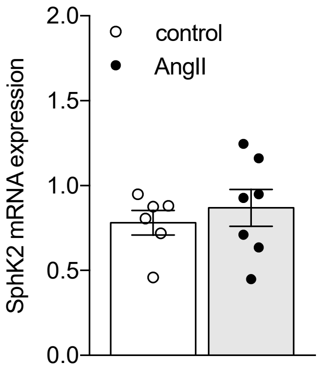

Figure S2. SphK2 expression remains unchanged in brain tissue of normotensive and hypertensive WT mice. qPCR analysis of *sphk2* mRNA expression of whole brain tissue from normotensive and hypertensive WT mice. Data expressed as mean ± SEM; $n = 6$ per group * $p < 0.05$ after single unpaired comparisons.

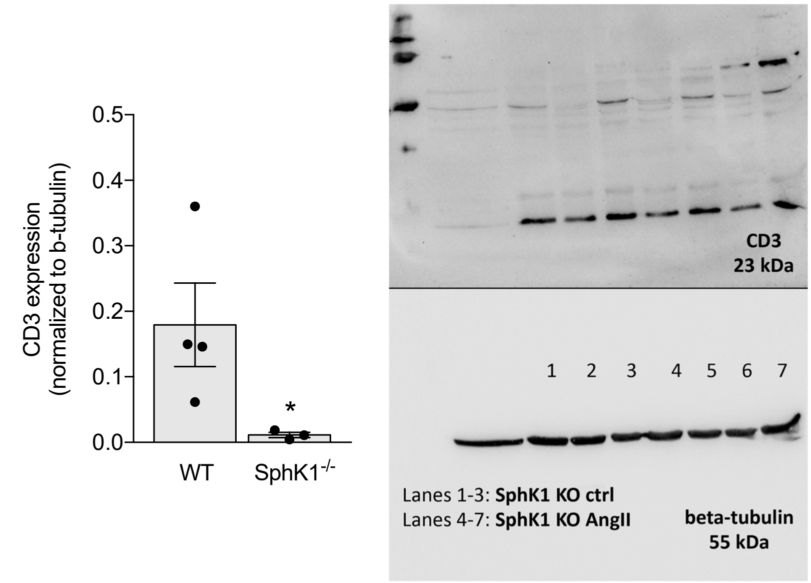

(**a**) (**b**)

Figure S3. Cerebral CD3 protein expression is significantly lower in SphK1$^{-/-}$ mice chronically treated with AngII compared to hypertensive WT mice. (**a**) Western blot analysis of CD3 in brain samples obtained from AngII-treated SphK1$^{-/-}$ mice and WT mice. Data expressed as mean ± SEM; n = 4 per group; * denotes $p < 0.05$ after single unpaired comparison. (**b**) Representative Western blot image illustrating unchanged CD3 protein expression in brain tissue lysates between control and AngII-treated SphK1$^{-/-}$ mice.

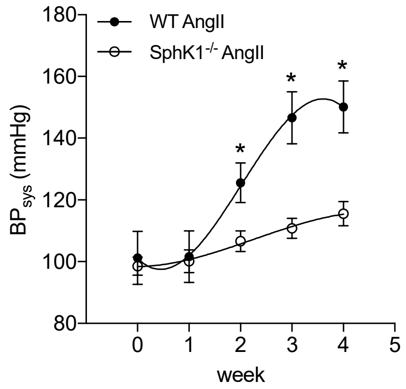

Figure S4. BP response to AngII differs between WT and SphK1$^{-/-}$ mice. Systolic BP to a four-week AngII treatment in WT and SphK1$^{-/-}$ mice. Data expressed as mean ± SEM; n = 6–7 per group. * $p <$ 0.05 relative to AngII-treated WT after 2-way repeated -measures ANOVA.

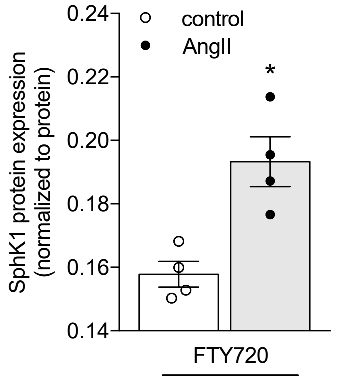

Figure S5. SphK1 protein expression increases in brain tissue of hypertensive WT mice treated with FTY720. Western blot analysis of SphK1 protein expression (after normalization with total protein) in

whole brain tissue from normotensive and hypertensive WT mice treated with FTY720 for two constitutive weeks. Data expressed as mean ± SEM; n = 4 per group * $p < 0.05$ after single unpaired comparisons.

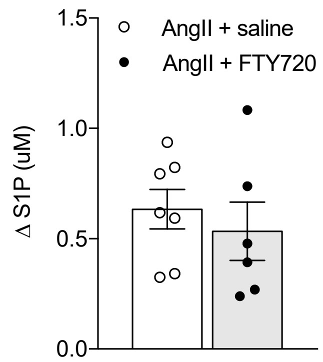

Figure S6. FTY720 does not affect the concentration difference between brain and plasma S1P levels (delta S1P) in hypertensive WT mice. Calculation of the difference between the S1P concentration in brain and plasma samples obtained by mass spectrometry from hypertensive WT mice treated with 1mg/kg BW FTY720 or placebo-treated hypertensive mice. Data expressed as mean ± SEM; n = 6–7 per group.

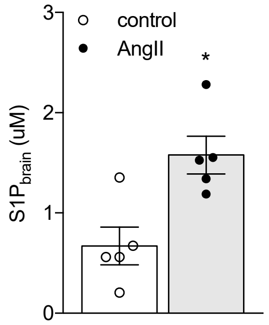

Figure S7. S1P concentration in brain tissue increases in Rag2$^{-/-}$ mice. Mass spectrometry analysis of S1P concentration in brain samples obtained from Rag2$^{-/-}$ mice chronically treated with AngII or saline. Data expressed as mean ± SEM; n = 5 per group * $p < 0.05$ after single unpaired comparisons.

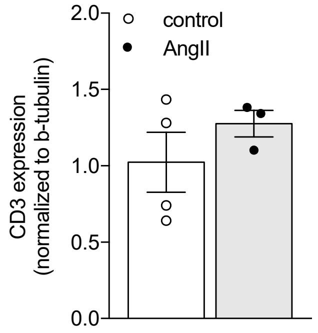

Figure S8. AngII treatment does not alter CD3 protein expression in brain tissue of Rag2$^{-/-}$ mice. Western blot analysis of CD3 in brain samples obtained from saline and AngII-treated Rag2$^{-/-}$ mice. Data expressed as mean ± SEM; n = 3-4 per group.

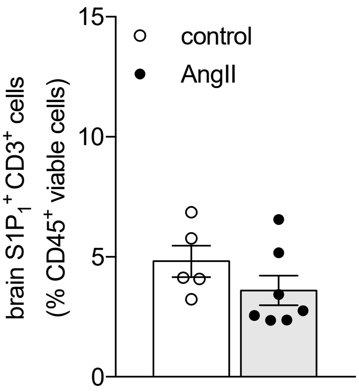

Figure S9. Hypertension does not affect the number of S1P1+ T-cells in the brain**.** Percentage of $S1P_1$+ CD3+ leukocytes in the brain of normotensive and hypertensive WT mice. Data expressed as mean ± SEM; *n* = 5–7 per group.

Table S3. Comparison of *sphk* transcript levels in brain tissue of WT and SphK1$^{-/-}$ mice. qPCR-based assessment of *sphk1* and *sphk2* transcript levels in brain tissue of different experimental groups. Data expressed as mean ± SEM; *n* = 6; * denotes *p < 0.05* after single unpaired comparisons between control and AngII within one group & # denotes *p < 0.05* after single unpaired comparisons between WT control and SphK1$^{-/-}$ control or WT+FTY720 control.

	sphk1		***sphk2***	
	**Control**	**AngII**	**Control**	**AngII**
WT	1.000 ± 0.038	1.318 ± 0.083 *	1.000 ± 0.058	1.040 ± 0.083
SphK1$^{-/-}$	0.003 ± 0.001	0.002 ± 0.001	1.875 ± 0.352 #	1.671 ± 0.222
WT + FTY720	1.571 ± 0.098	2.375 ± 0.312 *	1.571 ± 0.098 #	1.558 ± 0.158

Table S4. Circulating CD3+ T-cells in WT mice, SphK1$^{-/-}$ mice and WT mice treated with FTY720. FACS-based assessment of circulating CD45+ CD3+ cells in whole blood isolated from saline and AngII-treated SphK1$^{-/-}$ mice as well as normotensive and hypertensive WT mice subjected to two-weeks administration of FTY720. Data expressed as mean ± SEM; *n* = 6; *p* values calculated for single unpaired comparisons.

	**# CD45+ CD3+ Cells**	**% CD45+ CD3+ Cells**
WT control	35664 ± 3911	14.1 ± 0.90
WT AngII	81,264 ± 17,065	18.1 ± 0.91
p-value	*0.0254*	*0.0159*
SphK1$^{-/-}$ control SphK1$^{-/-}$ AngII	35,750 ± 8641<br>42,259 ± 6631	24.2 ± 1.84<br>25.56 ± 1.77
p-value	*0.2403*	*>0.999*
WT control + FTY720	3678 ± 884	6.59 ± 1.33
WT AngII + FTY720	843 ± 195	4.97 ± 0.55
p-value	*0.0303*	*0.3983*

Table S5. Comparison of chemokine and cytokine transcript levels in brain tissue of SphK1$^{-/-}$ and WT mice at baseline. qPCR-based assessment of pro-inflammatory chemokines and cytokines and endothelial activation markers in brain tissue of SphK1$^{-/-}$ and WT control mice. Data expressed as mean ± SEM; *n* = 6; *p* values calculated for single unpaired comparisons.

	**SphK1$^{-/-}$ Control vs. WT Control (Fold Change)**	***p*-Value**
Vcam1	1.07	*0.905*
Tnfa	1.05	*>0.999*
IL1b	1.25	*0.869*
Vwf	2.76	*0.016*
Selp	1.13	*>0.999*

Table S6. Gender-specific distribution of S1P concentrations in plasma and brain. Mass spectrometry-based assessment of S1P concentrations in plasma and brain tissue of all experimental groups separated according to gender. Data expressed as mean ± SEM.

	**Plasma S1P (μM)**		**Brain S1P (μM)**	
	**Control**	**AngII**	**CONTROL**	**AngII**
WT male (n = 4)	0.250 ± 0.042	0.560 ± 0.058	0.551 ± 0.048	1.036 ± 0.036
WT female (n = 3)	0.262 ± 0.232	0.506 ± 0.052	0.488 ± 0.047	1.271 ± 0.119
SphK1$^{-/-}$ male (n = 2)	0.078 ± 0.005	0.278 ± 0.098	0.567 ± 0.107	0.595 ± 0.004
SphK1$^{-/-}$ male (*n* = 3)	0.082 ± 0.027	0.167 ± 0.015	0.618 ± 0.149	0.561 ± 0.041
WT + FTY720 male (n = 3)	0.269 ± 0.004	0.518 ± 0.055	0.339 ± 0.016	0.921 ± 0.267
WT + FTY720 female (n = 3)	0.258 ± 0.027	0.551 ± 0.058	0.349 ± 0.024	0.846 ± 0.144
